# Nusinersen Wearing-Off in Adult 5q-Spinal Muscular Atrophy Patients

**DOI:** 10.3390/brainsci11030367

**Published:** 2021-03-13

**Authors:** Alma Osmanovic, Olivia Schreiber-Katz, Susanne Petri

**Affiliations:** Department of Neurology, Hannover Medical School, 30625 Hannover, Germany; schreiber-katz.olivia@mh-hannover.de (O.S.-K.); petri.susanne@mh-hannover.de (S.P.)

**Keywords:** spinal muscular atrophy, SMA, nusinersen, wearing-off, patient-reported outcome, antisense oligonucleotide, adult 5q-SMA patients

## Abstract

The antisense oligonucleotide nusinersen was the first drug treatment available for all types of 5q-spinal muscular atrophy (SMA). The dosing regime has been derived from pivotal clinical trials in infants and children. The efficacy of nusinersen in severely affected adult SMA patients is still questionable, as no placebo-controlled trials have been conducted. In the present study, we systematically examined wearing-off phenomena during nusinersen maintenance dosing using a patient-centered approach. We found that adult SMA patients perceived wearing-off after nearly half of 51 investigated nusinersen administrations, primarily within the last month prior to the next administration. Symptoms and functions affected were mainly general strength and arm and leg muscle function next to endurance and independence in daily routine. Lack of walking ability and higher body mass index were characteristic phenotypic features in patients with consistent wearing-off effects. We assume that specific SMA phenotypes might benefit from higher dosing, shorter treatment intervals, change of treatment administration or a combination of all. Efforts towards treatment optimization may result in higher efficacy in distinct phenotypes.

## 1. Introduction

Following the approval of nusinersen in December 2016, autosomal recessively inherited 5q-spinal muscular atrophy (SMA) caused by mutations in the survival of motor neuron 1 (*SMN1*) gene has become a treatable disease [1]. Nusinersen is an antisense oligonucleotide (ASO) which promotes SMN protein expression by interfering with SMN2 pre-mRNA splicing [2]. ASOs are short strands of synthetic nucleic acid that cannot cross the blood–brain barrier and have a limited half-life [3]. Therefore, nusinersen must be injected intrathecally and requires repeated lumbar punctures to maintain the pharmacological action [4]. Nusinersen dosing encompasses one persistent dose of 12 milligrams (mg) in 5 milliliters (mL), starting with four loading doses in between the first two treatment months, followed by maintenance doses once every four months [5]. Nusinersen has been approved for all 5q-SMA patients based on pivotal phase 3 trials exclusively conducted in infants and children, which showed a so-far never-seen improvement in motor function in this distinct patient group [6,7]. Although adults represent a relevant proportion of the overall SMA population, nusinersen tolerability, pharmacokinetics, safety and efficacy have not been investigated in adult SMA patients ahead of approval.

Only recently, nusinersen efficacy in adult SMA has been reported based on multicenter observational trials [8,9,10]. These studies demonstrated a clinically meaningful treatment effect, especially in less severely affected SMA patients, so far classified as SMA type 3. Type 3 patients are characterized by disease onset after 18 months of age and by reaching the motor milestone of being able to walk independently. This function can be lost during the disease course, so SMA type 3 comprises a broad clinical continuum [11]. SMA type 2 patients only acquire the ability to sit independently at some point, which may be lost with further disease progression [11]. They are assumed to benefit from nusinersen treatment; however, treatment effects are not sufficiently pictured by commonly used motor function outcome measures due to known floor effects and the lack of specific assessments addressing the needs of this patient group [8]. Therefore, patient-reported outcomes have been widely discussed to close this gap, and recent results confirm patients’ perceived efficacy even if non-ambulatory and severely affected [12,13,14]. Only recently, a study by Yeo CJJ et al. described two patients under regular nusinersen dosing reporting wearing-off phenomena. One patient indicated an increased reliance on his manual wheelchair and the return of fatigue in the month prior to the next maintenance dosing. The second patient, who had been non-ambulatory since adolescence, reported a less sufficient motor function regarding rise from supine to sitting position in the week preceding the next maintenance dose [15]. These effects occurred although both patients stated an overall subjective clinical improvement under nusinersen. A dose-dependent decline in nusinersen benefits between administrations was assumed.

Wearing-off phenomena are common in patients with Parkinson’s disease receiving levodopa therapy. Although they are generally thought to reflect the degeneration of presynaptic dopaminergic neurons, the underlying pathophysiology is proposed to be multifactorial. Wearing-off has a significant impact on patients’ quality of life and can be an important source of disability [16]. Therefore, understanding and minimizing wearing-off effects are important treatment implications.

The aim of this study was to further examine the only recently described fading effects of nusinersen between treatment intervals in adult SMA patients and to investigate the phenotypic features of the affected patients. Knowledge of the predictors of wearing-off phenomena will certainly lead to a better understanding of nusinersen treatment response and contribute to the ongoing discussion about the ideal nusinersen dose and administration frequency in adult SMA patients.

## 2. Materials and Methods

### 2.1. Study Design and Patient Characteristics

In a prospective single-center study, adult (≥18 years) 5q-SMA patients undergoing nusinersen treatment were enrolled between May 2020 and January 2021 at the Department of Neurology, Hannover Medical School, Germany. All patients willing to participate and able to perform the task were included in this study. At study enrolment, demographic as well as clinical characteristics, motor function status and treatment duration were assessed. Recorded parameters were age, sex, body mass index (BMI; weight in kilograms (kg), height in meters (m)), disease duration, SMA type, *SMN2* copy number, presence of scoliosis and administration route (computed tomography (CT)-guided or conventional lumbar puncture). Ambulatory state was defined as being able to walk at least ten meters without assistance or a device such as a cane or a walker [17]. Trained raters assessed two commonly used motor function measures in SMA. The Hammersmith Functional Motor Scale Expanded (HFMSE) consists of 33 items, scored on a scale from 0 to 2 with a total value of 66 points [18]. The HFMSE has been validated in type 2 and 3 SMA patients and measures gross motor functions such as rolling or sitting. The Revised Upper Limb Module (RULM) comprises 20 items with a maximum of 37 points, focusing on upper limb function [19].

This study was approved by the Ethics Committee of Hannover Medical School (No. 6269). Written informed consent was obtained from all subjects involved in the study before study enrolment.

### 2.2. Assessment of Treatment Response and Wearing-Off

As no validated questionnaire was available for this specific research question in SMA, a self-designed, standardized questionnaire was established to evaluate patients’ perceived wearing-off. The questionnaire consisted of six parts: (1) To assess whether or not patients had subjectively experienced a functional change under nusinersen treatment in general, a visual analog scale (VAS) was provided, ranging from 0 (much worse) over 5 (no change) to 10 (a lot better). Responses between 0 and 4 were rated as lack of treatment response; those rated as 5 (no change) up to 10 were rated as perceived treatment response. (2) Next, patients were asked to state if they had experienced a decrease in treatment effects in between two nusinersen dose deliveries, with a binary answer requested (yes or no) and the additional option to add a comment. (3) Patients who answered the previous question with “yes” were asked to rate the level of symptom deterioration compared to their condition after the last nusinersen administration. Patients were asked to choose the most appropriate value on a scale of 10–100% in 10% steps. (4) The fourth part dealt with the specific wearing-off regarding clinically meaningful symptoms or functions. Patients were asked to indicate these out of 18 items provided (1. Global strength; 2. Muscle strength of the legs; 3. Muscle strength of the arms; 4. Muscle strength of the trunk; 5. Endurance; 6. Mobility; 7. Pain; 8. Cramps; 9. Swallowing; 10. Speaking; 11. Respiratory symptoms; 12. Locomotion; 13. Independence in daily routine; 14. Falls; 15. Transfers; 16. Independent dressing; 17. Independent hygiene; 18. Independent eating). (5) Next, the time point when treatment effects started to wear off prior to the next nusinersen dosing was addressed. A scale was given, ranging from 1 week, 2 weeks, 3 weeks, 1 month, 2 months to 3 months. (6) At the end of the questionnaire, patients were asked “Do you think that this deterioration might result from wearing-off of nusinersen effectiveness?”. Again, a binary answer was requested (yes or no).

### 2.3. Statistical Analysis

Statistical analysis was conducted using IBM^®^ Statistical Software Package of Social Science (SPSS^®^, Chicago, IL, USA) version 26. Variables were summarized and provided in percent (%), as mean and standard deviation (SD) or median and range. Comparisons between groups were performed with the Mann–Whitney U test. Distributions of categorical variables were compared using the two-sided Fisher’s exact test. Statistical significance was two-tailed and set at *p* ≤ 0.05.

## 3. Results

Sixty-three questionnaires were completed by 31 SMA patients. Questionnaires that were filled in during the therapy loading period were excluded from the analysis since treatment intervals were short and no clinically meaningful treatment benefits were expected at this early treatment time point (*n* = 12; time since treatment initiation: two weeks *n* = 3; one month *n* = 4 and two months *n* = 5). As a result, 51 questionnaires were analyzed, filled in by 28 patients. Treatment duration ranged from 6 to 34 months. The recommended nusinersen treatment time points were met on average. The average deviation at enrolment was 2.63 (SD 10.10) days. Twenty-two patients filled in the questionnaire at least twice (two time points *n* = 21, three time points *n* = 1), whereas six patients were only assessed once. SMA type 2 and 3 patients were almost equally distributed (*n* = 13 and *n* = 14). Only one SMA type 4 patient was enrolled. Thirty-nine percent of patients were able to walk, and 50% suffered from scoliosis. The median age at enrolment was 36 years in this cohort, with a median disease duration of 33.8 years. The *SMN2* gene copy number ranged from two to six copies, with a median of three. The HFMSE at study enrolment was median 7.5 and ranged broadly from 0 to 66, and the RULM was median 21.0, ranging from 0 to 37 (Table 1).

### 3.1. Nusinersen Treatment Response and Wearing-Off

**Figure 1:**
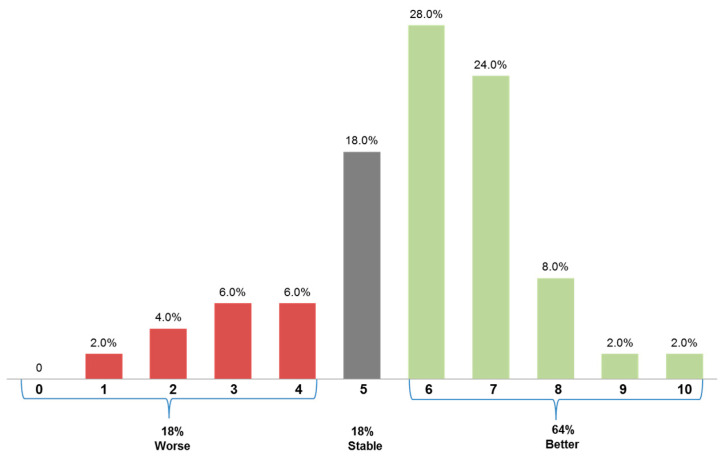
Patient-reported treatment responses. A visual analog scale demonstrates the patients’ perceived change in their overall condition, summarizing 51 nusinersen administrations. Zero marks “much worse”, 5 defines “no change” and 10 indicates an “a lot better” self-rated condition. Patients could choose their individual degree of either worsening of symptoms (red, 0–4) or improvement of symptoms (green, 6–10).

Symptom improvement under nusinersen treatment was reported following 64% of 51 nusinersen administrations, whereas a stable clinical condition and symptom deterioration were stated in 18% each (Figure 1). 

**Figure 2:**
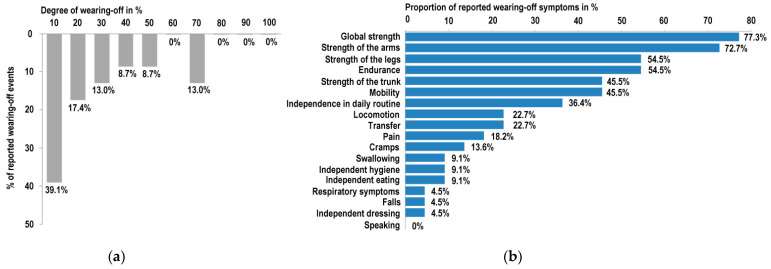
Quantitative and qualitative wearing-off effects subsequent to 51 nusinersen administrations. (**a**) In 23 (45.1%) of 51 nusinersen administrations, a decline in treatment effects was stated. The bar chart illustrates the degree of wearing-off symptoms between 10% and 100% compared to the individual condition after the last received nusinersen dosing. Predominantly, a mild wearing-off (10%) was reported, followed by 20% and 30% in 17.4% and 13.0% of cases, respectively. Rarely, a more severe deterioration (70%) was perceived. (**b**) Distribution of specific symptoms and functions affected by wearing-off. Strength, namely muscle function of the arms more than of the legs, endurance, mobility and independence in daily routine were mainly indicated to be affected by nusinersen wearing-off. Occurrences of pain and cramps were reported in 18.2% and 13.6%, respectively. Bulbar function (swallowing (9.1%) and speaking (0%)) does not seem to wane in between nusinersen administrations.

**Figure 3:**
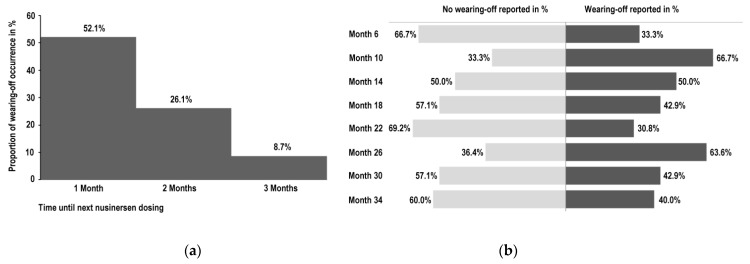
Assessment of time-dependency of nusinersen wearing-off subsequent to 51 nusinersen administrations. (**a**) The bar chart indicates the reported period prior to the next nusinersen dosing when symptoms or functions started to wane (*n* = 23). The last month before the next nusinersen administration was particularly at risk of wearing-off effects, confirming a time dependency. (**b**) Wearing-off phenomena were reported almost consistently during the treatment course. The displayed bar chart shows the ratio of reported wearing-off events (dark bar to the right) and no wearing-off phenomenon reported (light bar to the left) related to nusinersen treatment time points during maintenance dosing.

While in the majority of nusinersen administrations, a beneficial treatment response was reported, a gradual decline in treatment effects was stated subsequent to 23 (45.1%) of 51 nusinersen administrations. In 81%, this phenomenon was agreed to be likely related to a wearing-off. Furthermore, 11% disagreed and 8% did not answer this question. The individually experienced reduction in therapy efficacy in contrast to the effect immediately after the previous nusinersen administration was reported as follows: 10% reduction in 39.1% (*n* = 9), followed by 20% in 17.4% (*n* = 4) and 30% in 13.0% (*n* = 3) of 23 perceived wearing-off events. Only in 13.0% (*n* = 3) a major decline in efficacy of 70% was reported (Figure 2a). In the optional free text comment, patients mainly stated that their general motor function decreased nearly back to pre-treatment levels prior to the next nusinersen dosing. Regarding distinct functions affected by wearing-off, global strength and specifically strength of the arms were mostly affected, followed by muscle strength of the legs and trunk as well as endurance and mobility. Interestingly, in 36.4% of all perceived wearing-off symptoms, a reduction in independence in daily routine in advance of the next nusinersen dosing was reported. Bulbar function changes such as swallowing and speaking were less frequently affected by wearing-off (Figure 2b). The perceived deteriorations mainly occurred within the last month before the next nusinersen dosing (53%) (Figure 3a). During the therapy course, wearing-off was observed at any treatment time point. Hence, wearing-off phenomena could not be attributed to a distinct therapy duration (Figure 3b).

### 3.2. Phenotypic Features of SMA Patients with Perceived Wearing-Off

Twenty-three wearing-off events were reported by 16 of 28 (50.0%) enrolled patients. Seven of 28 patients (25.0%) repeatedly (at least twice) reported wearing-off phenomena, whereas eight of 28 patients (28.6%) consistently indicated no wearing-off effect at two independent time points during the treatment course. Seven patients (25.0%) were not consistent between two time points, and in a further six patients (21.4%), only one time point was available. To further characterize patients’ features associated with wearing-off effects, only patients consistently reporting a wearing-off phenomenon (*n* = 7) were compared to those patients who repeatedly reported no wearing-off at all (*n* = 8). No differences in sex, age or disease duration were identified (Table 2). However, a significantly higher BMI (mean 27.36 kg/m^2^, SD 5.07) was found in patients reporting wearing-off compared to those without wearing-off phenomena (mean 20.69 kg/m^2^, SD 5.08; *p* = 0.011). Furthermore, non-ambulatory patients were significantly more prevalent in the group of patients with wearing-off phenomena compared to those with no wearing-off (85.7% vs. 25.0%; *p* = 0.041). Other markers of disease severity (HFMSE, RULM, SMA type or *SMN2* copies, scoliosis) showed no significant differences. In addition, the grade of self-reported treatment effect did not vary between the two groups (mean 5.29 (SD 2.14) vs. mean 5.75 (SD 2.12); *p* = 0.766). Interestingly, patients who experienced a wearing-off tended to receive the next nusinersen dose a few days ahead of schedule compared to those with no wearing-off. However, this observation missed statistical significance (Table 2). 

## 4. Discussion

This study investigated the perceived phenomena of diminishing nusinersen treatment benefits over the four-month time interval between two administrations in adult 5q-SMA patients during maintenance dosing.

After nearly half of nusinersen administrations, patients reported a reduction in treatment effects, mainly by 10–30%, compared to their self-rated condition directly after the administration. This outcome was reported to emerge a few weeks ahead of their next dosing of nusinersen. By comparing patients who consistently reported this wearing-off phenomenon to those who did not experience any wane of effectiveness at all, patients with a higher BMI and without the ability to walk were identified to be at higher risk for nusinersen wearing-off.

ASOs such as nusinersen do not cross the blood–brain barrier and, thus, must be delivered by lumbar intrathecal injection [3]. Intrathecal administrations need to be repeated to maintain the maximal pharmacological efficacy due to drug clearance from the cerebrospinal fluid (CSF) and a designated half-life of four to six months [4]. Nusinersen promotes the expression and production of the missing full-length SMN protein by modifying the splicing of SMN2 pre-messenger RNA [3]. It can thereby improve disease symptoms rather than just slowing the progression of the disease [8]. As protein production is directly dependent on the presence of nusinersen and no self-regulating mechanisms exist on the molecular level, a reduction in drug concentration over time might reduce its consecutive clinical effects, resulting in the here-reported perceived wearing-off phenomena. 

Nusinersen gained approval based on two phase 3 trials, ENDEAR and CHERISH, which demonstrated its efficacy in infants and young children up to age 12 with SMA type 1 or 2 [6,7]. Unlimited approval of nusinersen in one dosing regime was given for all 5q-SMA patients regardless of age, size or disease severity, despite the lack of efficacy and dose-escalating trials in adult SMA patients [1].

Meanwhile, the efficacy of nusinersen in adult 5q-SMA patients has been demonstrated in large real-world multicenter studies [8,9]. Clinically meaningful motor function benefits were identified in less severely affected SMA type 3 patients. In adult SMA type 2 patients, clinically relevant treatment effects were more difficult to quantify, but at least disease stabilization has been shown [8,9].

Using a patient-centered approach to evaluate perceived treatment responses, our results match with these recently published data. In 64% of the investigated time points, a beneficial effect by nusinersen treatment, and in 18%, a stable clinical condition, was reported. In contrast, natural history data of adult SMA type 2 and 3 patients showed a slow but steady disease progression with decreasing muscle function and loss of motor milestones over time [20]. A potential beneficial treatment response, therefore, may be assumed in the majority of patients.

Despite highly prevalent treatment benefits, wearing-off phenomena within the treatment maintenance phase, with its four-month intervals between nusinersen administrations, were reported in 45% of received nusinersen administrations, independent of the overall treatment duration. This may raise the assumption that, although effective, the approved treatment regime might not be equally sufficient for all 5q-SMA patients. Nusinersen dosing was derived from phase 1 and 2 trials in infants and children. The open-label, phase 2, escalating-dose study assessed the pharmacokinetics, safety and tolerability and clinical efficacy of multiple intrathecal doses of nusinersen in two drug concentrations (6 and 12 mg) [5]. Twenty infant-onset SMA patients aged from three weeks to several months with a bodyweight of 5.1 to 9.3 kg were enrolled. Only the 12-mg dose group reached motor milestones and improvements in motor function scores compared to baseline. The beforehand performed open-label phase 1 trial investigated four increasing doses of nusinersen with a maximum of 9 mg per dose in 28 children (aged 2–14 years) [2]. Consistent with preclinical studies, CSF drug levels were found to be dose- and time-dependent. As indicated above, the current recommended nusinersen dosing is based on these two studies in infants and children and preclinical data focusing on 12 mg in a 5-mL volume, regardless of patient age and body weight. Due to the dose dependency, it may be possible that higher doses lead to increased clinical effects, but potential safety issues and off-target effects need to be taken into account. However, there are no reports published on higher doses yet, especially not in adult SMA patients. Yet, a clinical trial investigating this pivotal question is ongoing (DEVOT, https://clinicaltrials.gov/ct2/show/NCT04089566, accessed on 5 February 2021). Preliminary results are still pending.

Comparing patients repeatedly reporting wearing-off to those with no experienced diminishing effects, the BMI was significantly higher in patients with wearing-off phenomena. Interestingly, in a previous study, we already identified that patients with a higher BMI more frequently reported a deterioration of symptoms during the treatment course [21]. Higher BMI, however, is not associated to an increased CSF volume [22]. CSF volume rather correlates with age to a certain degree. CSF volume substantially increases from a few ml in infants to 150 ml by five years of age, without much increase further on [23]. Accordingly, no age-dependent wearing-off was identified in our adult cohort. However, it has been suggested that patients over two years of age have a reduced nusinersen distribution in the CSF relative to patients under two years of age, which were mainly included in the initial clinical trials [4]. Correspondingly, patients with the most robust SMN induction under nusinersen treatment in an autopsy cohort were between one and two years old at the time of death [24]. A lower CSF volume in infants and young children might, therefore, contribute to the known increased benefits of early and pre-symptomatic nusinersen treatment [25]. 

While BMI does not seem to change total CSF volume, it is, rather, associated to a decreased CSF volume in the lumbosacral region where intrathecal drugs are usually administered [26]. This might result in less sufficient nusinersen distribution in patients with higher BMI. Further studies need to investigate the impact of age and BMI on ASO drug distribution in the CSF.

Interestingly, non-ambulatory patients also reported significantly more wearing-off phenomena compared to ambulatory patients. A corresponding trend towards lower mean scores in motor function measures was seen (wearing-off: HFMSE = 17.67, RULM = 19.29 vs. no wearing-off: HFMSE = 33.25, RULM = 22.50), though not significant, presumably due to the low patient numbers assessed. Two potential reasons should be considered. As non-ambulatory SMA patients frequently suffer from severe scoliosis, a mechanically impaired distribution of nusinersen, especially towards more rostral areas, may be assumed [27]. This has already been demonstrated in patients with degenerative lumbar spinal stenosis [26]. However, there was no clear correlation of wearing-off phenomena with the presence of scoliosis in our study. As no severity grading, e.g., by Cobb angle, was performed, a possible effect of severe scoliosis cannot be excluded. Further investigations regarding CSF distribution of nusinersen in patients with severely deformed spines are urgently needed. Secondly, an area-dependent distribution of ASOs and nusinersen has previously been described and confirmed in vivo [3,24]. Herein, the most significant accumulation of SMN protein was identified in lumbar areas after intrathecal administration of nusinersen via lumbar puncture [24]. More rostral areas of the spinal cord, especially the cervical region, relevant for arm function, were found to have less SMN protein after lumbar drug delivery. In non-ambulatory SMA patients, arm or even only hand movements are the most important for their autonomy (e.g., use of a power wheelchair) and quality of life. Small changes due to wearing-off phenomena would be of high impact in these patients. This hypothesis is supported by the fact that decline in arm muscle strength was, besides reduction in general strength, the most frequently reported wearing-off phenomenon in this study.

Of note, in multicenter studies, nusinersen efficacy has, so far, mainly been measured by motor function outcome scores (HFMSE and RULM), which were performed on the day of nusinersen administration or the day after. Based on our data, potential fluctuations in nusinersen efficacy would only be detectable by an additional motor function investigation time point in between two subsequent nusinersen administrations.

As the majority of patients in our study did report some sort of individual improvement under therapy, our data lead to the suggestion that especially late-stage adult SMA patients may require a treatment optimization to reach a better nusinersen distribution and, thereby, more pronounced effects, even on upper limb function. This could possibly be achieved by a higher concentration of nusinersen, a higher volume or a more suitable delivery or, probably, a combination of all.

A higher dose, and therefore higher CSF drug levels, may potentiate nusinersen efficacy in patients who were described as non-responders in this study. It has already been reported that higher nusinersen CSF exposure correlated with greater increase in motor outcome scales as well as greater decline in neurofilament levels [28]. However, potential concordant increasing side effects need to be taken into account [2,10]. Interestingly, regarding other ASO strategies in neurological diseases, such as tofersen in amyotrophic lateral sclerosis (ALS), a drug dose-dependent effect on CSF protein concentration was also reported [29]. 

Besides dose escalation, an additional strategy would be to shorten treatment intervals. Our data showed that patients mainly reported a reduction in treatment effects during the fourth month. Patients in the wearing-off group even tended to arrange the subsequent nusinersen administration a few days earlier than expected compared to those who did not experience any wearing-off. An increase in treatment frequency would, however, increase the treatment burden for patients, as especially severely affected patients need special care and often CT-guided intrathecal drug administration. Furthermore, this strategy would significantly increase treatment costs from a societal perspective. Irrespective of whether a shorter interval might prevent wearing-off, patients should at least avoid treatment delays. 

Due to upcoming orally available splicing-modulating drugs for SMA, daily medication administration will become possible [30]. However, some off-target effects were discussed related to risdiplam [31]. Trials allowing for direct comparisons of intrathecally and orally administered splicing modulators are missing so far. In four years of clinical experience with nusinersen, any serious drug-related side effects have rarely been reported, although serious side effects regarding the administration procedure have occurred [10]. It is, therefore, to be discussed, though still highly speculative, whether combined advantages of both complementary splice-modifiers would be of specific benefit by synergistically enhancing the expression of the full-length SMN protein, especially in those patients with an apparently clinically meaningful effect under nusinersen treatment but wearing-off phenomena. Future investigations need to address this question.

This study has some limitations. SMA is a rare disease and nusinersen is an expensive treatment, which is only available at specialized neuromuscular centers in a multidisciplinary setting. Accordingly, only a rather small number of patients were included and analyzed in this study. The monocentric study design additionally led to small patient numbers. However, the main limitation of this study is the patient self-reported approach used to investigate wearing-off phenomena. Patients might have expected the treatment to be highly effective immediately after treatment and to lose efficacy over time until the next dose. A potential bias towards positive answers according to patients’ individual expectations cannot be excluded. Validation of these results, preferably by means of more objective outcome measures in a larger cohort, is warranted, especially regarding the further characterization of specific SMA phenotypes that might benefit from treatment modifications such as higher doses, shorter treatment intervals or a combination of two splice-modifying mechanisms. 

## 5. Conclusions 

This study identified clinically observed wearing-off phenomena in adult 5q-SMA patients under nusinersen treatment. Efforts towards treatment optimization in specific adult SMA patient phenotypes might improve the therapeutic efficacy.

## Figures and Tables

**Table 1:** Characteristics of enrolled adult 5q-spinal muscular atrophy (5q-SMA) patients.

*n* = 28	*n* (%)	Mean (SD)	Median (min.–max.)
Women	13 (46.4)		
Age * (y)		39.18 (12.90)	36.0 (20.0–66.0)
Disease duration * (y)		33.10 (13.67)	33.8 (3.0–64.5)
BMI *		22.06 (6.03)	22.5 (8.5–35.9)
Ambulatory	11 (39.3)		
Scoliosis	14 (50.0)		
CT-guided LP	16 (57.1)		
SMA type			
2	13 (46.4)		
3	14 (50.0)		
4	1 (3.6)		
*SMN2* gene copies			
2	2 (7.1)		
3	13 (46.4)		
4	10 (35.7)		
5	1 (3.6)		
6	2 (7.1)		
Motor function scores *			
HFMSE (max. 66)		25.08 (26.01)	7.5 (0–66)
RULM (max. 37)		20.77 (12.74)	21.0 (0–37)
Treatment duration * (m)		21.43 (7.75)	22.0 (6.0–34.0)
6 months	3 (5.9)		
10 months	3 (5.9)		
14 months	2 (3.9)		
18 months	7 (13.7)		
22 months	13 (25.5)		
26 months	11 (21.6)		
30 months	7 (13.7)		
34 months	5 (9.8)		
Discrepancy from dosing schedule (days) *		2.63 (10.1)	−1 (−11–35)
Count of treatment time points assessed per patient			
One time point	6 (21.4)		
≥Two time points	22 (78.6)		

* at study enrolment. Abbreviations: BMI, body mass index; CT, computed tomography; HFMSE, Hammersmith Functional Motor Scale Expanded; LP lumbar puncture; m, months; max., maximum; min. minimum; *n*, number; RULM, Revised Upper Limb Module; SD, standard deviation; SMN2, survival motor neuron 2; SMA, spinal muscular atrophy; y, years; %, percentage.

**Table 2:** Characteristics of SMA patients repeatedly reporting wearing-off vs. no perceived wearing-off phenomena.

	Wearing-Off (*n* = 7)		No Wearing-Off (*n* = 8)		*p*-Value
	*N* (%) or Mean (SD)	Median [min.–max.]	*N* (%) or Mean (SD)	Median [min.–max.]	
**Demographic**					
Women	2 (28.6%)		6 (75.0%)		0.132
Age (y)	38.86 (11.74)	35.0 [22.0-51.0]	42.63 (15.38)	36.5 [20.0-66.0]	0.524
BMI	27.36 (5.07)	25.95 [22.55-35.86]	20.69 (5.08)	20.69 [12.00-30.18]	0.011 *
**Clinical**					
Disease duration (y)	29.04 (16.71)	30.0 [2.8-50.0]	35.28 (18.12)	34.75 [3.0-64.5]	0.487
*SMN2* copies	3.43 (0.54)	3 [3-4]	4.00 (1.41)	4 [2-6]	0.421
SMA type	2.71 (0.76)	3 [2-4]	2.75 (0.46)	3 [2-3]	0.789
Non-ambulatory	6 (85.7%)		2 (25.0%)		0.041 *
Scoliosis	4 (57.1%)		3 (37.5%)		0.619
CT-guided LP	4 (57.1%)		4 (50.0%)		1.000
HFMSE (max. 66)	17.57 (25.51)	6.0 [0-66.0]	33.25 (27.69)	35.0 [1.0-64.0]	0.297
RULM (max. 37)	19.29 (11.49)	21.0 [1-7.0]	22.50 (13.52)	31.0 [5.0-37.0]	0.346
**Treatment**					
Treatment duration (months)	20.86 (6.41)	22.0 [10.0-30.0]	20.50 (6.02)	20.0 [10.0-30.0]	0.858
Schedule aberration (days)	−2.8 (6.0)	−2.0 [−13.5-5.0]	14.25 (27.97)	3.0 [−3.0-81.0]	0.072
VAS (0–10)	5.29 (2.14)	6 [1-7]	5.75 (2.12)	6 [2-9]	0.766

* *p*-value ≤ 0.05. Abbreviations: BMI, body mass index; CT, computed tomography; HFMSE, Hammersmith Functional Motor Scale Expanded; LP lumbar puncture; max., maximum; min., minimum; N, number; RULM, Revised Upper Limb Module; SD, standard deviation; SMN2, survival motor neuron 2; SMA, spinal muscular atrophy; y, years; %, percentage; VAS, visual analog scale regarding patients’ perceived change in their overall condition under nusinersen treatment (0= much worse to 10 = a lot better).

## Data Availability

All data generated or analyzed during this study are included in this published article.
